# Assessing the Knowledge of HPV-Associated Oropharyngeal Squamous Cell Carcinoma, HPV Vaccination, and Practice Scope among Saudi Dental Students in the Western Region

**DOI:** 10.3390/healthcare12090905

**Published:** 2024-04-26

**Authors:** Maha T. Alsharif, Elham Alsahafi

**Affiliations:** 1 Department of Oral Diagnostic Sciences, Faculty of Dentistry, King Abdulaziz University, Jeddah 21589, Saudi Arabia; 2 Department of Basic and Clinical Oral Sciences, Faculty of Dentistry, Umm Al-Qura University, Makkah 24382, Saudi Arabia; ensahafi@uqu.edu.sa

**Keywords:** HPV, oropharyngeal SCC, HPV vaccine, dental students

## Abstract

(1) Background: Human papillomavirus (HPV) infection is significantly associated with oropharyngeal squamous cell carcinoma (HPV-OPSCC), which is one of the fastest-growing cancer incidences globally. Dental practitioners play a crucial role in the primary and secondary prevention of HPV-OPSCC. There is little known about dental students’ awareness regarding HPV-OPSCC and HPV vaccination, as well as their intention to promote ‘primordial prevention’ among their patients. HPV vaccine, and the extent of their professional responsibilities. (2) Methods: This cross-sectional study was conducted in the western region of Saudi Arabia and involved undergraduate dental students (*n* = 688) from six public and private dental schools. Participants were requested to complete a sequential-section anonymous online survey, with 257 successfully completing all sections of the questionnaire. The association between participant characteristics and HPV-OPSCC, HPV vaccination awareness ratings, and perceived engagement in prevention were analyzed using ANOVA and chi-squared testing. A binary logistic regression analysis was conducted to examine the variables linked to these outcomes. (3) Results: Generally, the overall level of awareness of HPV-OPSCC and HPV vaccination was acceptable, with an average score of 53.44 ± 29.3 out of 100. However, a significant knowledge gap was observed, with 53% of respondents being unaware of the common sites for HPV-OPSCC and 63.8% being uninformed of the appropriate age for HPV vaccination. Females and those with a prior history of sexually transmitted diseases (STDs) had considerably higher levels of HPV vaccination knowledge (*p* < 0.05). The participants showed a favorable attitude towards their responsibility of informing patients about HPV-OPSCC and advocating HPV immunization. (4) Conclusions: This study underscores the need to enhance dental students’ understanding of HPV-OPSCC and HPV immunization, enabling them to effectively engage in primary and secondary preventative efforts against HPV-OPSCC.

## 1. Introduction

Oropharyngeal cancer (OPC), primarily squamous cell carcinomas (SCC) originating from the epithelial cell lining of the oropharynx, poses a serious worldwide health concern, with an estimated 99,000 new cases and over 48,000 deaths annually [1]. Emerging evidence has established a determinant role of high-risk human papillomavirus (HPV) infection (mainly HPV type 16 and HPV type 18) in the carcinogenesis of oropharyngeal squamous cell carcinomas (OPSCC) [2]. Therefore, the latest “WHO 2022” classification has recognized two types of OPSCC: HPV-associated OPSCC (HPV-OPSCC) and HPV-independent OPSCC, each with distinct epidemiological characteristics, clinical presentations, histopathological features, therapeutic approaches, and clinical outcomes [3]. Typically, HPV-OPSCCs are observed in younger, socioeconomically advantaged white males with increased sexual activity and minimal exposure to tobacco and alcohol, while HPV-independent OPSCCs are linked to older cohorts with lower socioeconomic status, chronic exposure to smoking and alcohol, and no association with increased sexual activity [4,5].

Furthermore, a more favorable prognosis is observed among HPV-OPSCC, better sensitivity to radiotherapy, and an improved overall survival rate when compared to HPV-independent OPSCC [6]. Over the past two decades, OPSCC incidence, driven by HPV-associated cases, has increased globally, particularly in developed countries such as the United States, where it will surpass the prevalence of cervical cancer. Mirroring trends have been observed in the United Kingdom and Europe [7,8,9]. Alternatively, in the Middle Eastern countries, including Saudi Arabia (SA), the prevalence of HPV-OPSCC is relatively lower [10], with investigations showing a low HPV prevalence among head and neck cancer (HNC) cases at around 21%, compared to global estimates ranging from 42.7 to 52.7% [7,11].

Despite the lower prevalence rate and better prognosis, HPV-OPSCC constitutes a significant portion of HNC cases in SA and is anticipated to become a major health burden [12], with primary tumors often hidden and diagnosed after metastasizing to cervical nodes [13]. Yet, specific screening methods or prevention measures are not available for OPSCC, unlike other HPV-related malignancies [14,15]. Thus, the potential effectiveness of the available HPV vaccines has been suggested for preventing HPV-OPSCC by the relevant authorities [16]. Recent studies indicate that patients primarily gain knowledge about HPV-OPSCC from their dentists, highlighting the crucial role of dentists in encouraging patients to seek information and ultimately preventing the progression of this condition [17,18,19]. The latest guidelines additionally advise dentists to actively promote HPV vaccinations, educate themselves and patients about HPV-OPSCC risk factors and symptoms, and conduct routine head and neck screenings [18].

Globally, the dental bachelor programs offer hands-on experience, with students actively engaging with patients in clinics. Thus, dental students’ knowledge of HPV-OPSCC is critical in identifying risk factors, prompting vaccination, and facilitating early intervention, thereby significantly contributing to public health initiatives aimed at reducing the burden of HPV-associated diseases [20]. In SA, previous studies identified a lack of HPV knowledge among dental students, necessitating curriculum enhancements, yet research on their understanding of HPV-OPSCC, attitudes toward HPV vaccination, and their roles in disease prevention and patient education remains limited [21,22]. Thus, this study aimed to (i) investigate the dental students’ knowledge of HPV-OPSCC and HPV vaccination in SA’s western region, and (ii) assess their attitudes toward their role in patient prevention and education. Lastly, this study aimed to (iii) determine the factors affecting dental students’ knowledge and attitude toward the HPV vaccine.

## 2. Materials and Methods

### 2.1. Study Design and Setting

This analytical cross-sectional study was conducted from January to August 2023. Undergraduate dental students and interns from six governmental and private dental schools located in the western region of SA were included in this study. Incomplete replies and first-year dental students, who were in their preparatory year, were excluded.

### 2.2. Sample Size and Participants Recruitments

The required sample size was calculated based on the population size of undergraduate dental students and interns in the selected universities (*n* = 1900) using a sample size calculator provided by Raosoft, Inc. (Seattle, WA, USA) (http://www.raosoft.com/samplesize.html) (accessed on 21 December 2022). Considering a 95% confidence level, 80% power, and a margin of error of 5%, the estimated sample size was (*n* = 320), which was increased to (*n* = 690) to account for non-responses and incomplete questionnaires. Students were recruited to participate in the study via representatives across each dental school, using a convenience sampling method. Dental students enrolled in levels 2 through 6 of the bachelor dental program, along with dental interns, were invited to participate in the study. Each class representative disseminated the survey using a direct link provided through the survey administration software (Forms, Google Inc., Mountain View, CA, USA, Version 0.8) via an instant messaging platform (WhatsApp, Facebook Inc., Menlo Park, CA, USA). Upon completion of the questionnaire, participants were instructed to submit the web form to the web server. Subsequently, the web server then transferred the data to a spreadsheet for analysis (Excel, Microsoft Corp., Albuquerque, NM, USA, Version 16.0).

### 2.3. Data Collection

The study questionnaire was adapted from previous studies [23,24]. The questionnaire was evaluated by two academic dental professionals to ensure content validity. Then, a pilot study was conducted involving 30 participants to assess question clarity and response time, and the results were not included in the final analysis. The Cronbach model was used to evaluate measurement scales, their items, and their average inter-item correlation. The questionnaire consisted of four main sections. The questionnaire was structured into four sequential sections to assess dental students’ knowledge of HPV-associated OPSCC and the HPV vaccine, as well as their perceived scope of practice. The first section collected information about participants’ characteristics and covariables, including gender, marital status, dental school, year of enrollment (second years to interns), and grade point average (GPA). Participants were also asked about their history of sexually transmitted diseases and smoking habits.

**Figure 1:**
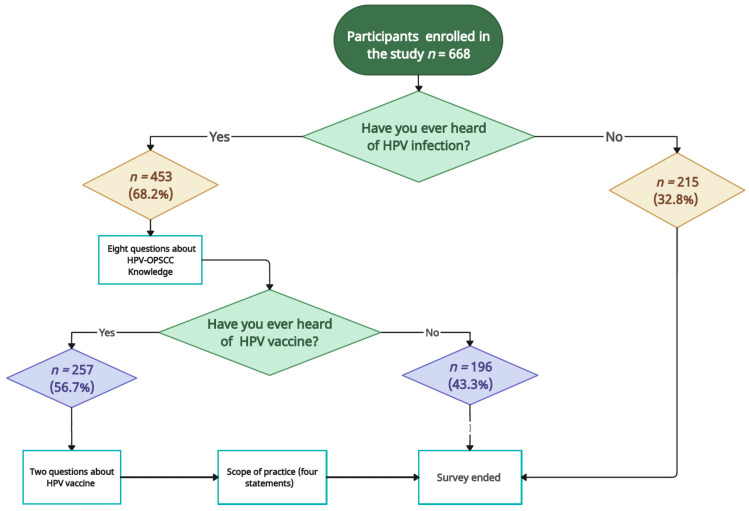
Flowchart illustrating the sample collection process based on participants’ responses.

At the end of the first section, a screening question was posed to ascertain awareness of HPV: “Have you heard about HPV?”. A positive response of “yes” directed participants to the second section, which included eight general HPV-OPSCC self-perceived knowledge questions (yes/no), followed by a multiple-choice question regarding the sources from which the participants obtained their information regarding HPV-OPSCC. A negative response of “no” concluded the questionnaire. The third section included the screening question regarding awareness of the HPV vaccine: “Have you heard about the HPV vaccine? Participants affirming familiarity with the HPV vaccine were then guided to the third section, which included two HPV vaccination self-perceived knowledge questions (yes/no) and a multiple-choice question about the source of HPV vaccination knowledge. By answering all questions in Section 3, the participants were subsequently moved to the fourth and final section. It consisted of four 5-point Likert scale statements about the scope of practice (1 = strongly disagree; 5 = strongly agree). In this section, participants were asked about their agreement about whether the patients’ education and awareness about HPV-OPSCC, recommending HPV vaccines, and attending continuous education courses fall within their scope of practice. Figure 1 illustrates the flowchart of the questionnaire answering process.

### 2.4. Ethical Consideration and Informed Consent

Ethical approval was granted by the research ethics committees at Umm Al-Qura University Dental College in Makkah, SA (approval no. HAPO-02-K-012-2022-11-1257) and King Abdulaziz University in Jeddah, SA (approval no. 123-10-22).

The study employed an anonymous, closed-ended questionnaire in English, the primary language of instruction in dental schools. A cover page that explained the study’s objectives, the voluntary nature of participation, the confidentiality protocols for the gathered data, and the approval obtained from the institutional review board was included. Additionally, contact information for the researchers was provided in case participants had further inquiries. Approximately 20 min were needed to complete the questionnaire. Participants consented electronically by agreeing to answer the questionnaire questions.

### 2.5. Statistical Analysis

Data were analyzed using IBM SPSS version 27 (IBM Corp., Armonk, NY, USA) and visually presented by GraphPad Prism version 8 (GraphPad Software, Inc., San Diego, CA, USA). Categorical and nominal variables were described by counts and percentages, whereas continuous variables were described by mean and standard deviation. Two scores were calculated: “HPV knowledge” and “HPV scope of practice”. Each “HPV knowledge” question was transformed to 1 and 0 for correct and incorrect responses. A simple additive algorithm and a 100-point scale were used to determine the score. Reliability analysis with a Cronbach model was used to evaluate measurement scales, their items, and their average inter-item correlation. To correlate scores, which were both represented by means, Pearson’s correlation coefficient was used.

To assess the association between the scores and demographic information, we conducted an independent *t*-test when comparing two group means, and for scenarios involving more than two groups, a one-way ANOVA was employed. Additionally, post hoc testing was performed using the least significant difference (LSD) method. These tests were done with the assumption of a normal distribution. Otherwise, the Games–Howell test for multiple groups was used as an alternative for the LSD test. To establish associations among categorical variables, this investigation employed the chi-squared test. The conventional threshold for rejecting the null hypothesis was set at a *p*-value less than 0.05. The study considered the dependent variables as binary outcomes. Binary logistic regression modeling, utilizing backward conditional elimination with entry criteria set at 0.05 and elimination criteria at 0.10, was employed to identify significant predictors of the dependent variables, each reported with 95% confidence intervals. Furthermore, a general linear model univariate analysis was conducted to identify significant predictors using the main effect as the model. Once again, the conventional *p*-value threshold of <0.05 was used to reject the null hypothesis.

## 3. Results

### 3.1. HPV-OPSCC Knowledge

Out of the total participants (*n* = 668) who began the survey and completed the first section, only 68% (*n* = 453) demonstrated awareness of HPV infection and were directed to the next section’s HPV-OPSCC knowledge questions. On the other hand, the majority of participants who had not heard about HPV (*n* = 215) were females (18%), single (31%), had no history of sexually transmitted diseases (STDs) (31%), and were enrolled in preclinical years (23%) (Supplementary Table S1). HPV knowledge, including vaccination knowledge, was evaluated using a score scale ranging from 0 to 100. The average HPV-OPSCC score was 53.44 (SD ± 29.3), which was satisfactory (Table 1). Similarly, the scope of practice was evaluated using a score system ranging from 1 to 16. The average scope of practice score was 12.16 (SD ± 3), which was satisfactory (Table 1). The characteristics of study participants and their association with HPV knowledge scores, vaccine awareness, and scope of practice are summarized in Table 1. Table 2 summarizes the responses of participants (*n* = 453) to HPV knowledge questions, including HPV vaccination questions, as well as their assessments.

In order to assess the reliability of our study’s data, a Cronbach’s alpha test was conducted to correlate the scores of participants’ HPV knowledge with their scope of practice.

The result indicated that the data pertaining to participants’ knowledge of HPV-OPSCC (0.651) and their scope of practice (0.797) exhibited satisfactory reliability (Supplementary Table S2). Furthermore, a statistically significant correlation was seen between these two variables (*p* < 0.001) (Supplementary Table S2). The relationship between participants’ characteristics and their knowledge of HPV-OPSCC demonstrated comparable increased knowledge scores across both male and female participants. Furthermore, older dental students demonstrated significantly higher HPV-OPSCC knowledge scores than younger dental students (*p* < 0.012) (Table 1). Also, there was a strong association between higher knowledge scores and participants who were in clinical years and had better GPA grades (*p*< 0.001 and *p* < 0.047) in comparison to those in non-clinical years and lower GPA who had lower HPV knowledge scores (Table 1). The majority of study participants acquired their HPV knowledge from dental education (53%), social media platforms (21%), and health care providers (10%) (Supplementary Figure S1).

### 3.2. HPV Vaccine Knowledge

**Figure 2:**
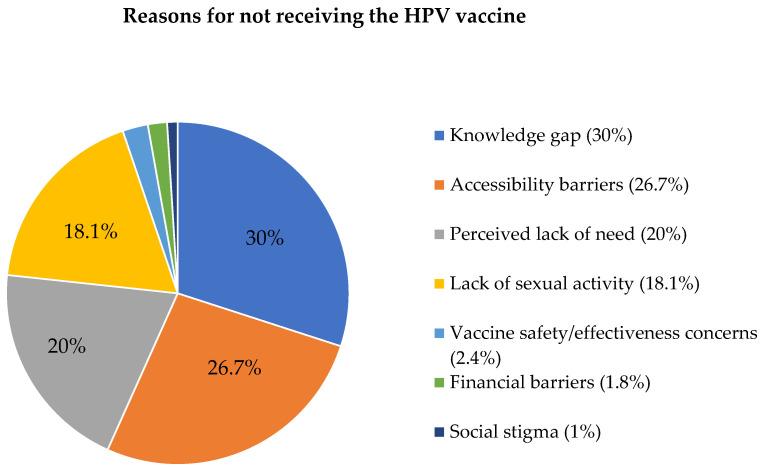
Reasons for not receiving the HPV vaccine.

**Figure 3:**
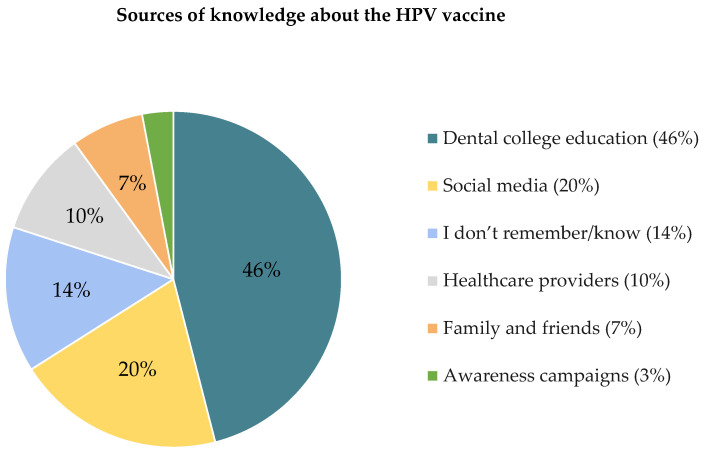
Sources of knowledge about the HPV vaccine.

Given that knowledge about HPV significantly influences vaccine awareness and acceptance, we investigated participants’ awareness of the HPV vaccine, including the appropriate age for vaccination, reasons for vaccine hesitancy, and their sources of information. Although (*n =* 257) of participants indicated awareness of the HPV vaccine, a significant majority, 63.8% (*n =* 164), showed a lack of knowledge on the appropriate age for HPV vaccination (Supplementary Table S3). Moreover, a majority of participants who were aware of the HPV vaccine were not vaccinated, 76.7% (*n =* 197) (Supplementary Table S3). The main factors contributing to this trend are a lack of HPV vaccine knowledge (30.5%) and uncertainty about vaccination facilities (26.7%). Also, 20% of participants perceived a lack of need, and 18.1% cited their lack of sexual activity as a reason for not getting vaccinated (Figure 2). Participants primarily acquired knowledge about the HPV vaccination through dental education (46.1%), while only 20% reported learning about it through social media platforms. Notably, 14% of participants were unable to recall the source of their knowledge about the HPV vaccination (Figure 3).

There was a significant association between the characteristics of participants and their awareness of the HPV vaccination (Table 1). Specifically, a gender-based analysis revealed that a larger percentage of female participants were aware of the HPV vaccine compared to male participants (*p* < 0.018) (Table 1). The history of STDs played a notable role in influencing knowledge regarding HPV vaccination, as indicated by statistical significance (*p* < 0.008) (Table 1). The association between participants’ HPV vaccine knowledge and their HPV knowledge scores is demonstrated in Table 3. The findings demonstrated a strong association between higher HPV knowledge scores and vaccination awareness among participants (*p* < 0.031) (Table 3). Lower HPV knowledge scores were shown to be associated with a lack of understanding of the recommended age for HPV vaccination among participants (*p* < 0.001) (Table 3).

### 3.3. HPV in the Dental Practice Scope

Participants demonstrated a generally positive attitude toward incorporating HPV knowledge and vaccine awareness into their professional responsibilities, as participants have shown a keen interest in educating their patients about HPV and advocating for the delivery of the HPV vaccination. In addition, the participants showed a positive attitude toward actively participating in continuing professional development (CPD) courses and HPV awareness programs, as seen by the data given in Supplementary Table S4. To correlate the favorable attitude of respondents with their HPV knowledge scores, a statistically significant (*p* < 0.001) correlation was observed, as higher HPV knowledge scores were associated with positive attitudes of respondents, as shown in Supplementary Table S2. The association between participants’ characteristics and their attitude toward HPV within their field of practice is presented in Table 1.

### 3.4. Regression Model Analysis

Regression analysis was performed to assess the degree of connection between HPV knowledge, HPV vaccination, and participants’ characteristics (Table 4). In regard to this study dependent variable (HPV knowledge), age and GPA were significant predictors for higher HPV knowledge, as the age group (25–30) was predicted to pose higher HPV knowledge scores (*p* < 0.018) (Table 4). Similarly, a higher GPA (4.4–4.9) was predicted to have a higher HPV knowledge score (*p* < 0.006) (Table 4). For the other study’s dependent variable (HPV vaccine knowledge), history of STDs was a significant predictor of higher HPV vaccine knowledge (*p* < 0.015) (Table 4).

## 4. Discussion

The burden of HPV-OPSCC is growing globally, and it is suggested to be potentially preventable by the available HPV vaccines [25]. Healthcare practitioners’ primary and secondary prevention may reduce the burden of HPV-OPSCC. Moreover, assessing knowledge, attitude, practices, and readiness regarding HPV-associated OPSCC and its prevention for future healthcare providers must be continuously assessed and upgraded before entering the workforce. Therefore, this study aimed to assess the knowledge, attitudes, and practices of dental students in the western region of Saudi Arabia regarding HPV-OPSCC and the HPV vaccine.

Our study is the first to be conducted in the western region of Saudi Arabia, and it involved all governmental and private dental colleges in the region. Previous studies focused solely on other regions of Saudi Arabia or a single city in the western region [26,27,28]. In this study, participants demonstrated acceptable HPV awareness (mean score of 53.44 ± 29.3 out of 100), with the majority recognizing HPV-OPSCC as a sexually transmitted disease. On the other hand, 32% had not heard of HPV infection, with the majority being preclinical students (23%) with no prior experience with STDs (31%).

The undergraduate students’ baseline HPV knowledge is a significant focus since it will boost their contribution to preventing HPV-OPSCC. Our findings demonstrated better baseline HPV awareness among participants than in a previous study by Farsi NJ et al., which only included participants from one dental school in the western region and found low HPV knowledge scores [23]. Furthermore, our participants’ HPV awareness scores are comparable to a study by Lingam et al. that found generally sufficient HPV knowledge among dental students from numerous Asian and African countries [29]. In contrast, inadequate levels of HPV-related knowledge, HPV-OPSCC awareness, and HPV vaccination were reported among American oral health students [30]. This global diversity emphasizes the importance of understanding regional variations in knowledge and attitudes. Our findings revealed that clinical students had much higher HPV-OPSCC knowledge than those in preclinical years. This outcome is predicted since clinical students are more likely to encounter patients with oral diseases, particularly HPV-related ones, and receive more hands-on experience. Furthermore, it was assumed that those students were further along in their studies, having completed courses in oral pathology and oral cancer prior to conducting the survey. Furthermore, the distribution of clinical (61.8%) and preclinical (38.2%) students may have influenced HPV knowledge scores. Previous research indicated that clinical students were more aware of HPV infection and linked malignancies than preclinical students, emphasizing the value of direct patient-care experience and advanced coursework [23]. Furthermore, we discovered that higher GPAs are a significant predictor of higher HPV awareness, which was not predicted in prior studies in the western region. According to this association, academically successful or motivated students may be more motivated to acquire the necessary knowledge. A similar pattern was observed in an Australian study, which found that dental students with superior academic performance had a greater understanding of oral health conditions [31].

Only 56.7% of our study’s participants demonstrated awareness of the HPV vaccine, while 43.3% were unaware of it, reflecting a significant HPV vaccine knowledge gap among our participants. Addressing this disparity is critical because insufficient HPV vaccine knowledge might lead to ineffective patient education. However, our findings indicated a higher level of HPV vaccine knowledge compared to only 45% of American dentists in a multi-state study that acknowledged enough knowledge level about HPV vaccination [31]. In the United Kingdom, dental students presented a much higher awareness level, with 90% being knowledgeable about HPV vaccination [32]. These disparities may be explained by global differences in HPV knowledge teaching in the dental curriculum, national vaccination policies, and cultural variables [33]. Therefore, a recent Malaysian study revealed that incorporating HPV and its vaccination knowledge into dental curricula resulted in more knowledgeable and informed dental practitioners [34].

As a result, we identified a statistically significant association between better HPV knowledge scores and HPV vaccine awareness among participants (*p* < 0.031). Also, female participants showed significantly higher levels of HPV vaccine knowledge than their male counterparts (*p* < 0.018). This could be attributed to the fact that HPV-related cancers primarily affect women, making them more likely to learn and presumably recall more HPV-related information. This is consistent with previous studies, which demonstrated significant gender disparities in HPV vaccination awareness [35]. Regardless of the perceived risk to one gender, the dental curriculum should encourage HPV vaccination for all genders. It should also eliminate the false belief that only women need HPV immunizations and that only women develop HPV cancer. Similar to our findings, Ahken et al. reported that HPV vaccination awareness is substantially related to STDs in young Canadian women [36]. While previous research found no link between STDs and HPV awareness [23], our findings are the first in the region to show that having a personal history of STDs is a robust predictor of greater HPV vaccine awareness. These findings support location-based health education for a variety of demographics. Female dental students were found to be more knowledgeable in Saudi Arabia, emphasizing the importance of targeted instructional strategies to effectively reach male dental students.

In terms of the HPV vaccination rate, only 23% (*n* = 60 out of 257) reported being vaccinated, which is considered to be inadequate but significantly higher than Farsi et al.’s vaccination rate of 16% among Saudi dental students [23]. The Saudi Ministry of Health’s (MoH) 2021 strategic decision to include HPV vaccination on the national schedule might be responsible for the 44% rise in vaccination rates. However, this vaccination rate remains low, indicating trends in other regions, particularly developing nations. For instance, a report by Chiang et al. indicated a 13% rate of HPV vaccination among university students in Hong Kong, while Rashwan et al. found a 3% vaccination rate among Malaysian medical students [34,37]. Research showed that healthcare practitioners’ HPV vaccination promotion is connected to their own vaccine worries and views, supporting a “learning effect” (the more self-experienced they are, the more likely they are to promote vaccination) [38].

The study participants’ lack of HPV vaccination stemmed primarily from two key factors: a deficiency in awareness regarding the vaccine (30%) and uncertainty regarding its availability (26.7%). This lack of HPV vaccination requirements among participants could be attributed to limited dental curriculum coverage or the later inclusion of the HPV vaccine in the Saudi national vaccination program in 2021. A notable portion of unvaccinated individuals (18.1%) held the belief that the HPV vaccine lacked efficacy before the onset of sexual activity. This perception may be rooted in a dearth of information, coupled with cultural beliefs in Saudi Arabia, particularly the stigma surrounding sexual activity, which could hinder access to accurate information [39]. In addition, only 36% knew the age of the HPV vaccine. Because the vaccine is only effective for both genders between the ages of nine and twelve, a lack of information may impede patient advocacy for HPV vaccination. This disparity could be attributed to a lack of HPV knowledge, college exposure to HPV-related information, and increased social, cultural, and religious issues [40].

Dental education was the primary source of HPV vaccine knowledge for more than half of the participants (46%). Furthermore, social media was used as a secondary source of HPV vaccine information (20%). Thus, our study mirrored a previously documented shift in learning preferences among today’s students, who are increasingly reliant on media [41]. As a result, it is suggested that traditional academic sources be combined with media channels to improve HPV education. These findings revealed that knowing about the vaccine alone was insufficient, and comprehensive HPV-OPSCC and vaccination knowledge in the dental curriculum should be implemented to improve dental students’ capacity to motivate patients and deliver correct information.

One of the study’s significant findings is a strong link between HPV awareness and scope of practice. Our participants acknowledged HPV-OPSCC and HPV vaccine awareness and prevention, in accordance with the Ministry of Health’s national strategy and ADA standards. Thus, dental students must be prepared to respond to patient inquiries. Our participants also expressed a desire to improve their roles by pursuing continuing dental education courses. Other studies found that participants thought HPV knowledge was outside their scope of practice and should be left to other healthcare providers [42]. Thus, future research should evaluate what influences dentists’ HPV-OPSCC and vaccination advice. Identifying factors connected to lower HPV awareness can help the program decide whether dentists need HPV training. Our study regression model revealed that key predictors that influence HPV knowledge and HPV vaccine awareness are higher GPA, older students in clinical years, and a history of STDs, as indicated by our study data.

### Strengths and Limitations

Our study’s strengths include being the first to survey all dental schools in Saudi Arabia’s western region, both public and private, and obtaining a high response rate, which increased the sample’s variety and representativeness. However, caution should be considered when extrapolating our findings to other dental institutions in different SA regions. While previous studies in Saudi Arabia focused on HPV knowledge and vaccination among healthcare students, ours was also the first to investigate their perceived role in HPV-OPSCC prevention.

Certain shortcomings in our study require that they be addressed. First, there is the possibility of selection bias because those who actively engage may have a certain level of prior knowledge about HPV, which may not correctly reflect the characteristics of the whole student population. Second, the usage of online surveys may have an impact on the accuracy of knowledge levels, as participants may seek answers while completing the survey. Third, it is crucial to note that the lack of predetermined cutoff values for assessing HPV knowledge scores in the study group may alter the interpretation of our data.

## 5. Conclusions

Under the present MoH immunization strategy, this study provides insight into the knowledge, attitudes, and practices of dental students in the western region of Saudi Arabia on HPV-OPSCC and HPV vaccination. Notably, participants showed diverse levels of knowledge about HPV and its risks, with the majority recognizing HPV-OPSCC as a sexually transmitted disease. However, considerable knowledge gaps remained, especially among preclinical students and those with no prior experience with sexually transmitted diseases. Despite improvements in HPV vaccine awareness compared to previous studies, a substantial knowledge gap regarding the HPV vaccine, the optimal age for HPV vaccination, and its efficacy persisted among participants.

These findings highlight the critical need for efforts to improve educational dental curricula, focusing knowledge gaps about HPV-OPSCC, HPV vaccination, and its implications for oral health, with the goal of preparing them to be on the front lines of primary and secondary prevention of HPV-related oral cancers. The current study’s findings are also promising since dental students had an overall favorable attitude toward their involvement in HPV prevention and stated a motivation to continually improve their expertise in this sector.

## Figures and Tables

**Table 1:** Association of participants’ characteristics with HPV knowledge scores, vaccine awareness, and scope of practice scores.

Demographics		Total *n* (%)	HPV Knowledge Score	*p*-Value	HPV Vaccine Awareness		*p*-Value	Total *n* (%)	HPV Scope of Practice	*p*-Value
					Yes *n* (%)	No *n* (%)				
Total		453 (100%)	53.44 ± 29.3	-	257 (56.7%)	196 (43.3%)	-	257 (100%)	12.16 ± 3.0	-
Age (years)	<20	25 (5.5%)	42.80 ± 32.2 ^AC^*	0.012 ^a,c^	12 (48.0%)	13 (52.0%)	0.23	12 (4.7%)	10.08 ± 2.7	0.101
	20–25	364 (80.3%)	54.84 ± 28.9 ^B^*		203 (55.8%)	161 (44.2%)		203 (79%)	12.25 ± 3.0	
	25–30	61 (13.5%)	51.64 ± 29.1 ^AB^*		41 (67.2%)	2 (32.8%)		41 (16%)	12.32 ± 2.8	
	>30	3 (0.7%)	10.00 ± 10.0 ^C^*		1 (33.3%)	2 (66.7%)		1 (0.3%)	12.00 ± 0.0	
Gender	Male	198 (43.7%)	54.75 ± 30.2	0.405	100 (50.5%)	98 (49.5%)	0.018 ^d^	100 (38.9%)	12.20 ± 3.0	0.848
	Female	255 (56.3%)	52.43 ± 28.6		157 (61.6%)	98 (38.4%)		157 (61.1%)	12.13 ± 2.9	
Dental college	Private	160 (35.4%)	55.82 ± 27.1	<0.008 ^b^	101 (63.1%)	59 (36.9%)	0.043 ^d^	101 (39.3%)	12.25 ± 3.0	<0.098
	Governmental	293 (64.6%)	48.97 ± 26.1		156 (53.2%)	137 (46.8%)		156 (60.7%)	11.7 ± 2.3	
Clinical	Clinical	353 (77.9%)	56.29 ± 29.1	<0.001 ^b^	201 (56.9%)	152 (43.1%)	0.867	201 (78.9%)	12.26 ± 3.0	0.268
	Non-Clinical	100 (22.1%)	43.40 ± 27.9		56 (56.0%)	44 (44.0%)		56 (21.1%)	11.77 ± 2.8	
Grade point average	≤2.99	8 (1.8%)	47.50 ± 27.1 ^AB^*	0.047 ^a,c^	6 (75.0%)	2 (25.0%)	0.83	6 (2.3%)	10.00 ± 2.5	0.068
	3–3.49	46 (10.1%)	49.35 ± 30.7 ^AB^*		25 (54.3%)	21 (45.7%)		25 (9.7%)	12.12 ± 2.2	
	3.50–3.99	81 (17.9%)	45.80 ± 32.6 ^B^*		48 (59.3%)	33 (40.7%)		48 (18.7%)	12.58 ± 3.2	
	4–4.49	155 (34.2%)	55.35 ± 28.7 ^A^*		87 (56.1%)	68 (43.9%)		87 (33.9%)	12.60 ± 2.8	
	≥4.50	163 (36%)	56.87 ± 27.3 ^A^*		91 (55.8%)	7 (44.2%)		91 (35.4%)	11.66 ± 3.1	
Marital status	Single	425 (93.8%)	53.34 ± 29.1	0.772	243 (57.2%)	182 (42.8%)	0.458	243 (94.6%)	12.21 ± 2.9	0.221
	Married	28 (6.2%)	55.00 ± 32.4		14 (50.0%)	14 (50.0%)		14 (5.4%)	11.21 ± 3.7	
History of sexually transmitted disease	Yes	17 (3.8%)	65.29 ± 33.2	0.089	15 (88.2%)	2 (11.8%)	0.008 ^d^	15 (5.8%)	12.33 ± 2.1	0.811
	No	436 (96.2%)	52.98 ± 29.1		242 (55.5%)	194 (44.5%)		242 (94.2%)	12.14 ± 3.0	
Smoking status	Yes (current/former)	127 (28%)	56.61 ± 30.3	0.151	65 (51.2%)	62 (48.8%)	0.137	65 (25.3%)	12.43 ± 2.7	0.386
	No	326 (72%)	52.21 ± 28.8		192 (58.9%)	134 (41.1%)		192 (74.7%)	12.06 ± 3.0	

^a^—Significant using one-way ANOVA test at <0.05 level. ^b^—Significant using the independent *t*-test at the <0.05 level. ^c^—Post hoc test = LSD. ^d^—Significant using the chi-square test at the <0.05 level. * Significant relationships between subgroups were identified using post hoc analysis and subgroups with different letters denote statistically significant differences (*p* < 0.05).

**Table 2:** Assessment of participants’ knowledge of HPV-OPSCC and the HPV vaccine.

HPV, HPV-OPSCC and Vaccine Knowledge Questions	Assessment of HPV Infection and HPV-OPSCC Knowledge *n* (%)	
	Correct	Incorrect
Understanding of HPV types (low-risk and high-risk)	280 (61.8%)	173 (38.2%)
HPV’s link to cervical cancer and OPSCC	282 (62.3%)	171 (37.7%)
High-risk HPV link (HPV type 16) to OPSCC	226 (49.9%)	227 (50.1%)
Global Increase in HPV-Associated OPSCC Prevalence	203 (44.8%)	250 (55.2%)
HPV-OPSCC has clear clinical signs and symptoms	215 (47.5%)	238 (52.5%)
Improved prognosis for HPV-OPSCC	177 (39.1%)	276 (60.9%)
Spread of HPV infection	329 (72.6%)	124 (27.4%)
Common sites for the onset of HPV-OPSCC	213 (47.0%)	240 (53.0%)
HPV vaccine awareness	257 (56.7%)	196 (43.3%)
Suitable age for the HPV vaccine *	164 (63.8%)	93 (36.2%)

* Responses limited to participants aware of the HPV vaccine; *n* = 257.

**Table 3:** Association between participants’ HPV vaccine knowledge and HPV-OPSCC knowledge scores.

HPV Vaccine Knowledge		Total *n* (%)	HPV-OPSCC Knowledge	*p*-Value
Awareness of the HPV vaccine (*n* = 453)	Yes	257 (56.7%)	56.03 ± 28.6	0.031 ^a^
	No	196 (43.3%)	50.05 ± 30.0	
Received HPV vaccination previously (*n* = 257)	Yes	60 (23.3%)	59.33 ± 28.5	0.307
	No	197 (76.7%)	55.03 ± 28.6	
Suitable age for the HPV vaccine (*n* = 257)	Yes	93 (36.2%)	67.53 ± 27.6	0.001 ^a^
	No	164 (63.8%)	49.51 ± 27.1	

^a^—Significant using the independent *t*-test at the <0.05 level.

**Table 4:** Regression analysis and parameter estimates.

	Dependent Variable: HPV Knowledge						
	Parameter	B	S.E.	95% C.I.			*p*-Value
				Lower Bound		Upper Bound	
	Intercept	4.838	15.197	−25.031		34.706	0.750
Age	<20	44.899	16.875	11.733		78.065	0.008 ^a^
	20–25	44.944	15.670	14.145		75.742	0.004 ^a^
	25–30	37.966	15.931	6.654		69.277	0.018 ^a^
Grade point average	<2.99	−9.802	10.069	−29.591		9.987	0.331
	3–3.49	−7.943	4.781	−17.340		1.454	0.097
	3.50–3.99	−10.082	3.699	−17.351		−2.812	0.007 ^a^
	4.4–4.9	−8.590	3.107	−14.697		−2.482	0.006 ^a^
	**Dependent Variable: HPV Vaccine Knowledge**						
	**Variables in Equation**	**B**	**S.E.**	**Exp (B)**	**95% C.I. for Exp (B)**		***p*-Value**
					**Lower**	**Upper**	
Step 1 ^b^	Gender (Male)	0.479	0.194	1.614	1.104	2.360	0.013 ^c^
	History of sexually transmitted disease (Yes)	−1.859	0.762	0.156	0.035	0.694	0.015 ^c^
	Constant	−0.431	0.130	0.650			0.001 ^c^

^a^—Significant using a general linear model at the <0.05 level. ^b^—Variable(s) entered on step 1: gender, positive history of sexually transmitted disease. ^c^—Significant using the binary logistic regression model, with backward conditional elimination with Enter Criteria = 0.05 and Elimination = 0.10.

## Data Availability

The raw data supporting the conclusions of this article will be made available by the authors on request.

## Supplementary Materials

The following supporting information can be downloaded at: https://www.mdpi.com/article/10.3390/healthcare12090905/s1, Figure S1: Sources of knowledge about the HPV; Table S1: Characteristics of study’s participants and their awareness of Human papillomavirus (HPV) infection (*n* = 668); Table S2: Reliability and dependability of study main domains (HPV knowledge and HPV Scope of Practice) and correlation; Table S3: HPV vaccine knowledge assessment; Table S4: HPV Scope of Practice scores assessment.
