# Supplementary material for: Assessing the Knowledge of HPV-Associated Oropharyngeal Squamous Cell Carcinoma, HPV Vaccination, and Practice Scope among Saudi Dental Students in the Western Region
Source: Healthcare (Basel). 2024 Apr 26;12(9):905. doi: 10.3390/healthcare12090905 (PMC11083101; doi:10.3390/healthcare12090905)
Supplement: Supplementary file 1 [file healthcare-12-00905-s001.zip › healthcare-2932365-supplementary.pdf]

Table S1 (Appendix): Characteristics of study's participants and their awareness of Human papillomavirus (HPV) infection (N=668)

Demographics		HPV infection awareness n (%) *		P-value
		Yes	No	
Total		453(67.8%)	215(32.2%)	-
Age (years)	< 20	25(3.7%)	59(8.8%)	0.001*
	20-25	364(54.5%)	141(21%)	
	26-30	64(9.4%)	15(2.3%)	
Gender	Male	198 (29.6%)	93(14%)	.912
	Female	255 (38.2%)	122(18%)	
Dental College	Private	160 (24%)	74(11%)	.82
	Governmental	293 (44%)	141(21%)	
Academic level	Clinical	353 (53%)	60(9%)	0.001*
	Non-Clinical	100 (15%)	155(23%)	
GPA (out of 5)	≤ 2.99	8 (1.2%)	11(1.6%)	.152
	3-3.49	46 (6.6%)	25(4%)	
	3.50-3.99	81 (12%)	36(5.4%)	
	4-4.49	155(23%)	66(10%)	
	≥4.50	163(24.2%)	77(12%)	
Marital status	Single	425(64%)	204(31%)	.583
	Married	28(4%)	11(2%)	
History of sexually transmitted disease	Yes	17(2.5%)	7(1.5%)	.75
	No	436(65%)	208(31%)	
Smoking status	Yes	127(19%)	41(6%)	.013*
	No	326(49%)	174(26%)	

Chi-square test, Significant at p<0.05

Table S2 (Appendix): Reliability and dependability of study main domains (HPV knowledge and HPV Scope of Practice)

**Reliability Statistics**	**Cronbach's Alpha**	**N of Items**
HPV knowledge	0.651	10
HPV scope of practice	0.797	4

Figure S1 (appendix)

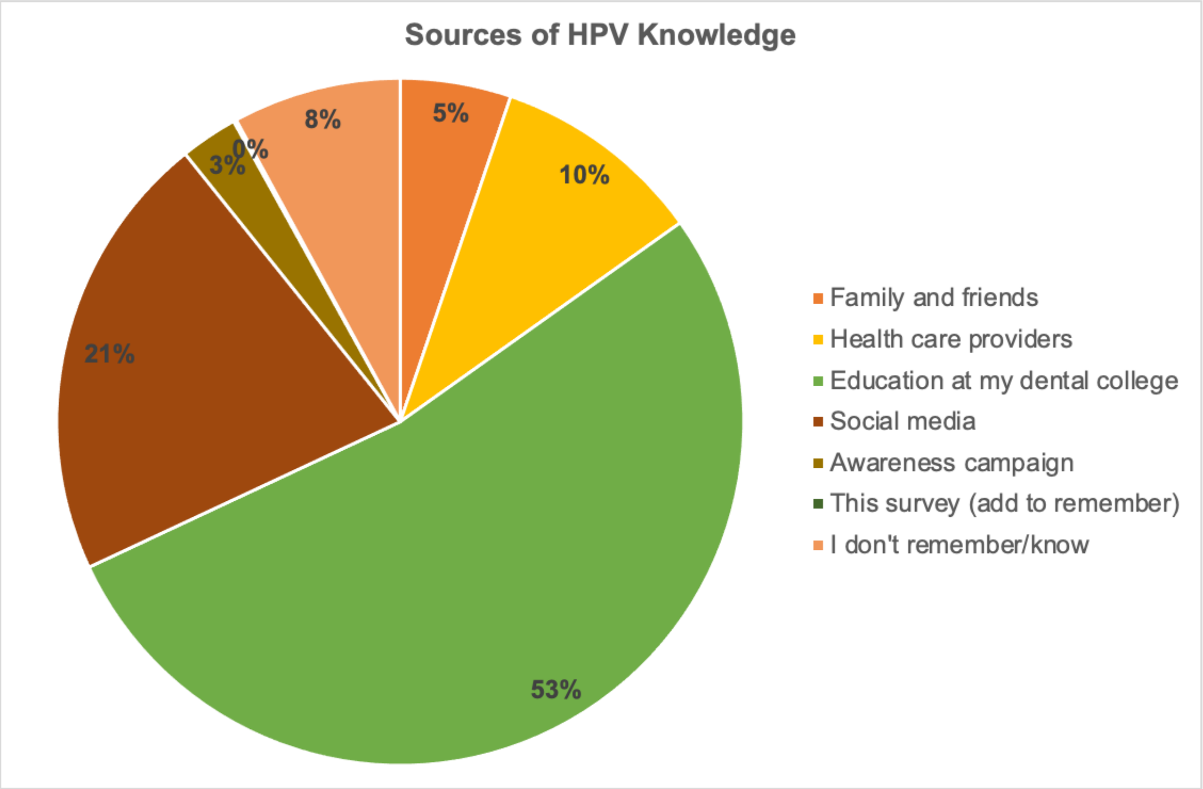

Figure S1. Sources of knowledge about the HPV

Table S3 (Appendix): HPV vaccine knowledge assessment.

		N	%
Total		453	100.0
Have you ever heard of the human papillomavirus (HPV) vaccine?	Yes	257	56.7
	No	196	43.3
**Variables**		**N**	**%**
Total		257	100.0
Do you know the adequate age to get the vaccine?	Yes	93	36.2
	No	164	63.8
Are you vaccinated against HPV?	Yes	60	23.3
	No	197	76.7

Table S4 (Appendix): HPV Scope of Practice scores assessment

HPV scope of practice	N	Min	Max	Mean	SD
Discussing the link between HPV and oropharyngeal cancer with the patients falls within the scope and role of the dental professional	257	0.00	4.00	3.09	0.9
Recommending HPV vaccination with the patients falls within the scope and role of the dental professional	257	0.00	4.00	2.95	1.0
Attending continuing educational courses about HPV role in OPSCC falls within the scope and role of the dental professional	257	0.00	4.00	3.11	0.9
Planning public awareness campaigns about HPV infection, vaccination and OPSCC falls within the scope and role of the dental professional	257	0.00	4.00	3.01	1.0
